# Computational Assessment of Facial Expression Production in ASD Children

**DOI:** 10.3390/s18113993

**Published:** 2018-11-16

**Authors:** Marco Leo, Pierluigi Carcagnì, Cosimo Distante, Paolo Spagnolo, Pier Luigi Mazzeo, Anna Chiara Rosato, Serena Petrocchi, Chiara Pellegrino, Annalisa Levante, Filomena De Lumè, Flavia Lecciso

**Affiliations:** 1 Institute of Applied Sciences and Intelligent Systems, National Research Council of Italy, via Monteroni, 73100 Lecce, Italy; pierluigi.carcagni@cnr.it (P.C.); cosimo.distante@cnr.it (C.D.); paolo.spagnolo@cnr.it (P.S.); pierluigi.mazzeo@cnr.it (P.L.M.); 2 Amici di Nico Onlus, Via Campania, 6, 73046 Lecce, Italy; annachiara.rosato@libero.it; 3 USI, Institute of Communication and Health, Via Buffi 6, 6900 Lugano, Switzerland; serena.petrocchi@usi.ch; 4 L’ Adelfia Onlus, via S. Sangiovanni, 115-73031 Lecce, Italy; chiara.pellegrino@yahoo.it; 5 Dipartimento di Storia, University of Salento, Società e Studi Sull’ Uomo, Studium 2000-Edificio 5-Via di Valesio, 73100 Lecce, Italy; annalisa.levante@unisalento.it (A.L.); filomena.delume@unisalento.it (F.D.L.); flavia.lecciso@unisalento.it (F.L.)

**Keywords:** quantitative facial expression analysis, geometrical and temporal regularization of facial action units, ASD diagnosis and assessment

## Abstract

In this paper, a computational approach is proposed and put into practice to assess the capability of children having had diagnosed Autism Spectrum Disorders (ASD) to produce facial expressions. The proposed approach is based on computer vision components working on sequence of images acquired by an off-the-shelf camera in unconstrained conditions. Action unit intensities are estimated by analyzing local appearance and then both temporal and geometrical relationships, learned by Convolutional Neural Networks, are exploited to regularize gathered estimates. To cope with stereotyped movements and to highlight even subtle voluntary movements of facial muscles, a personalized and contextual statistical modeling of non-emotional face is formulated and used as a reference. Experimental results demonstrate how the proposed pipeline can improve the analysis of facial expressions produced by ASD children. A comparison of system’s outputs with the evaluations performed by psychologists, on the same group of ASD children, makes evident how the performed quantitative analysis of children’s abilities helps to go beyond the traditional qualitative ASD assessment/diagnosis protocols, whose outcomes are affected by human limitations in observing and understanding multi-cues behaviors such as facial expressions.

## 1. Introduction

Automatic analysis of facial actions is being gathering incremental interest in the computer vision community [1]. This interest is evidenced by the continuous introduction of new computational methods deploying the most advanced theories in automatic learning and recognition of patterns. Very comprehensive surveys on this topic are in [2,3] where also affect-related applications are listed.

Very outstanding approaches in this arena are those based on complex deep learning architectures [4,5] and generative models [6,7] that, by exploiting knowledge extracted from many data [8], have reached the highest accuracy on benchmark datasets.

At the same time, effective approaches using engineered representations [9], such as Gabor motion energy, Local Binary Patterns or Local Phase Quantisation, and dynamic representations [10] have also been proposed.

However, despite progress in new approaches as well as benchmarking efforts, most evaluations still focus on either posed expressions, near-frontal recordings, or both. This makes it hard to evaluate their ecological validity, i.e., to exploit them in real application scenarios where they have to work under unconstrained conditions [11].

Besides, works in the state of the art mainly focus on qualitative recognition of facial expressions, i.e., they are designed to make decisions concerning if either a given facial expression is present or not [12]. Typically, they give as output one of six (or seven if also the neutral one is contemplated) basic expressions together with a classification confidence of each expression [13]. It follows that they do not take care of the challenging issue of automatically providing a quantitative assessment of facial expression production. Automatic quantitative assessment of facial expression can be defined as the ability of a system to analyze facial cues and to give a numerical outcome describing how much the produced facial expression is similar to the expected one in an objective way. The lack of standard rules for expression intensity labeling and the limited availability of labeled data are the two most drawbacks in this research area that includes only a few recent pioneering works limited to a single expression [14], making use of 3D data [15] or requiring unrealistic constrained evolutions such as an initial neutral stage (with no expression) followed by the onset of expression ending with expression apex [16].

Consequently, computer vision for getting computational face expression analysis is not exploited yet in real scenarios such as those related to the diagnosis and assess of neurological or physical diseases [17]. On the other side, this emerging application area, referred to as behavioral imaging [18], affective computing [19] or sentiment analysis [20], is very promising and one of its most interesting research topics concerns the design of smart environments for the computational study of children with Autism Spectrum Disorders (ASD) [21].

Psychological works have, in fact, established links between children with ASD and difficulty in producing and perceiving emotional facial expressions [22]. Facial expressions in children with ASD are notably distinct from those in typically developing children and are difficult to be detected by visual inspection. Facial expression production mechanisms in autism involve understanding the overall dynamics of the entire face, localized dynamics in specific facial regions, as well as the dynamical relationships between the movements across different facial regions [23]. The milestone of the use of behavioral imaging for autism analysis is the work in [24]. Afterward, a plethora of works dealt with automatic discovering the differences between the production of facial expressions in ASD and non-ASD groups even by acquiring multimodal response data [25].

To the best of our knowledge, there are no published works exploiting computer vision to quantitatively assess the ability of children with ASD to produce facial expressions. Invasive methods (based on eye tracker and electroencephalography) [26] or questionnaire measures [27] have been successfully applied to this aim instead, providing scientific proofs that the production of expressions can be strengthened through practice and training.

Unfortunately, in therapy with children, both the invasive tools and the questionnaires become unusable and therefore the only viable path is the evaluation performed by observers but, of course, it is unelectable biased by their interpretation and emotional state [28]. On the other side, the observer’s perception is also influenced by the children’s face appearance and by the mutual motion of the facial regions that can alter the sensitivity to micro-expressions [29]. This is a very challenging aspect, as demonstrated by the efforts that some authors did to partially control noisy distortions of the ground truth by statistical strategies based on modeling multiple time series annotations [30].

In this paper, a computational approach, based on computer vision components working on sequences of images acquired by an off-the-shelf camera in unconstrained conditions, is proposed and evaluated to assess the capability of children with ASD to produce facial expressions. The main contributions of the paper can be summarized as follows:

- A new processing pipeline based on computer vision methods able to analyze facial expressions in unconstrained conditions is introduced is presented.
- Action unit intensities are estimated by analyzing local appearance and then both temporal and geometrical relationships, learned by Convolutional Neural Networks, are exploited to regularize gathered estimates.
- A statistical modeling of non-emotional face configurations, able to embed stereotyped movements and to highlight even subtle voluntary movements of facial muscles, is formulated.
- It experimentally demonstrates how the proposed pipeline can improve the analysis of facial expressions produced by ASD children.
- It reports a comparison of system outputs with respect to the evaluations performed by professionals on the same group of ASD children. This makes evident how the proposed pipeline could help to go beyond the limitations of the traditional ASD assessment/diagnosis protocols whose outcomes are affected by human limitations in observing and understanding multi-cues behaviors such as facial expressions.
- It provides new and attractive perspectives to exploit computer vision to monitor the evolution of children’s skills over time to objectively highlight the improvements (for example, to compare the individual skills before and after targeted therapies).

The rest of the paper is organized as follows. In Section 2, the processing pipeline based on computer vision and statistics methods is presented. Section 3 assesses the pipeline on a publicly available dataset. Section 4 reports experimental evidences of the advantages in using the proposed pipeline to quantitatively analyze the ability of children with ASD to produce basic facial expressions. Finally, Section 5 concludes the paper and gives a glimpse about possible research lines to pursue in future works.

## 2. System Overview

**Figure 1:**
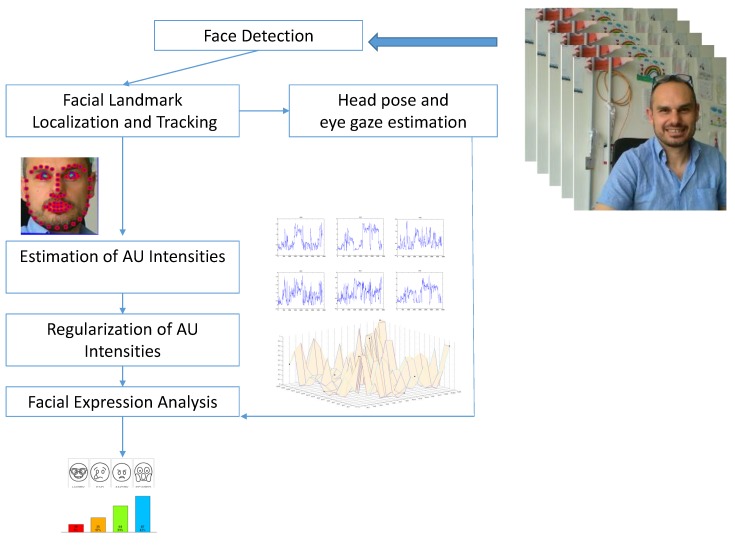
The algorithmic pipeline.

The system works on image sequences acquired from off-the-shelf cameras. The algorithmic pipeline consists of five main components: a face detector, a facial landmark detection and tracking block, a head pose estimation and eye gaze tracking block, a facial action unit intensity estimator and a high-level semantic data analysis module. Each component is detailed in the following. A schematic representation of the pipeline is shown in Figure 1.

Face detection is performed by using Histogram of Oriented Gradients (HOG) feature within a sliding window framework [31]. In particular, the HOG filter used is learned via Max-Margin Object Detection (MMOD), as recently proposed in [32], and implemented in the dlib computer vision library (http://dlib.net/) to improve face detection. The MMOD uses a set of images *X* and associated labels *Y* and it attempts to find the best parameters such that the detector makes the correct prediction on each training sample. The cutting plane method, which iteratively refines a feasible set or objective function by means of linear inequalities, is then used to solve the related Max-Margin Object Detection optimization problem. The learned filter has size of 80×80 pixel. By upsampling each image by a factor of two, it is possible to detect smaller faces, i.e., face that are larger than about 40×40 pixels in size. To perform detection, this single HOG filter is slid over the image at each level of an image pyramid. This way no subsampling and parameter manipulation is required. The method finds the set of sliding window positions which pass a threshold test. To handle the removal of overlapping bounding boxes (that refer to the same object), non-maximum suppression practice, on the Mean-Shift algorithm, is applied.

Facial landmark detection and tracking is carried out by an extension of the original Conditional Local Neural Field (CLNF) proposed in [33]. CLNF is an instance of the Constrained Local Models (CLM) proposed in [34] and consists of two main components:

- A Point Distribution Model (PDM) aimed to model the location of facial feature points in the image using non-rigid shape and rigid global transformation parameters.
- Patch experts deployed to capture appearance of local patches around landmarks.

A CLM model can be described by a set of parameters p=[s,R,q,t] that can be varied in order to acquire various instances of the model: the scale factor *s*; object rotation R (first two rows of a 3D rotation matrix); 2D translation t; and a vector describing non-rigid variation of shape q. The point distribution model (PDM) is: $x_{i}=s\cdot R\left({\bar{x}}_{i}+\Phi_{i}q\right)+t$. Here, $x_{i}=\left(x,y\right)$ denotes the 2D location of the *i*th feature point in an image, ${\bar{x}}_{i}=\left(X,Y,Z\right)$ is the mean value of the *i*th element of the PDM in the 3D reference frame, and the vector $\Phi_{i}$ is the *i*th eigenvector obtained from the training set that describes the linear variations of non-rigid shape of this feature point.

In CLM (and CLNF), the maximum a posteriori probability (MAP) of the face model parameters p given an initial location of the parameters determined by a face detection step is estimated.

The solution in use improves the standard approach by means the training of separate sets of point distributions and patch expert models for eyes, lips and eyebrows. As a successive step, it fits the landmarks detected with individual models to a joint PDM. The tracking phase is supported by a face validation step aimed to avoid face leading or face drifting over a long period of time. To this end, the system employs a Convolutional Neural Network (CNN) that, given a face aligned using a piece-wise affine warp, predicts the expected landmark detection error. In this way, the models can be reset when the validation step fails. As a final enforcement, a multiple initialization hypotheses (at different orientations) is employed to pick the best convergence likelihood and manage challenging situations such as faces acquired in the wild.

The used PDM (36 non-rigid and 6 rigid shape parameters) and CNN are both trained on the LFPW [35] and Helen [36] training sets. On the other hand, the CLNF patch experts are trained on Multi-PIE [37], LFPW [35] and Helen [36] training sets. A key point for the robustness of the proposed approach is the use of 28 sets of patch experts trained at different scales and views that allow handling different images resolution of the face under analysis, as well as head rotations and consequent self occlusions. The CLNF model is initialized with the face detector provided by the dlib library [38] whose bounding box is linearly mapped to the one surrounding the 68 facial landmarks.

The adopted solution gives accurate estimation on head pose and eye gaze by exploiting the information provided by the CLNF. It employs a 3D representation of facial landmarks (projected on the image using an orthographic camera projection) that allows the evaluation of head pose orientation. The role of camera calibration parameters for an accurate head pose estimation that is anyway kept strong enough if based on the rough estimation of the image size is straightforward.

Detection of eye regions landmarks (eyelids, iris and pupils) is done generalizing the generic CLNF deformable shape registration approach to the specific eye regions problem. More precisely, the PDM model and CLNF patch experts have been trained on the SynthesEyes training dataset [39]. The obtained CLNF eyes model is then used in order to detect the location of the eye and the pupil and, consequently, to compute the eye gaze vector individually for each eye. A ray is fired from the camera origin through the center of the pupil in the image plane and the intersection with the eye-ball sphere is computed leading to the pupil location in 3D camera coordinates. Finally, the vector from the 3D eyeball center to the pupil location is the estimated gaze vector.

Estimation of Action Unit Intensities is the following step in the implemented pipeline. The reliability of an action unit classifier depends largely on the employed training data and its ability to estimate facial expressions of a subject when his neutral one is unknown. The proposed solution exploits the idea proposed in [40] where the authors introduced a real-time Facial Action Unit intensity estimation and occurrence detection system based on geometry features (shape parameters and landmark locations computed by the CLNF) and appearance (Histograms of Oriented Gradients). Firstly, the detected face is mapped onto a common reference frame. To this end, the currently detected landmarks are transformed to a representation of frontal landmark from a natural expression (a projection of mean shape from a 3D PDM). This results in a 112×112 pixel image of the face with 45 pixel interpupilary distance. To remove non-facial information from the image, a masking of the image is performed using a convex hull surrounding the aligned feature points. The aligned face results in a 112×112 image ready for appearance features extraction. In this step, Histograms of Oriented Gradients (HOGs) are extracted as proposed in [41]. Blocks of 2×2 cells, of 8×8 pixels are employed and lead to 12×12 blocks of 31-dimensional histograms. The final vector size is of 4464 elements describing the face subsequently reduced to 1391 elements by means of a Principal Component Analysis (PCA) approach. The non-rigid shape parameters and landmark locations in object space inferred during CLNF model tracking are used as geometry based features that results in a 227-dimensional vector. The complete features vector is then made up by the concatenation of the geometry and appearance ones. To account for personal differences, the median value of the features is subtracted from the estimates in the current frame. Finally, the AU intensities are estimated by Support Vector Machines (SVM) and Support Vector Regression (SVR) employing linear kernels. The models used in the proposed approach are trained on DISFA [42], SEMAINE [43] and BP4D [44] datasets. By using the distance to the hyperplane of the trained SVM model as a feature for an SVR regressor, it was possible to gather information from the above non-overlapping datasets (this allowed overcoming the problem that BP4D contains only information about AU’s presence).

A Regularization of AU Intensities is then necessary due to the nature of the gathered estimates. The AU intensities estimated as described above rely indeed only on “static” (i.e., frame based) appearance models built by concatenating the patch experts in the CNLF and the HOG features. This results in an independent estimation of each AU intensity that is ineluctably affected by errors (especially in the case of images acquired in unconstrained conditions) that come also from unknown sources by making them hard to be prevented. It is straightforward to observe that there are temporal and spatial relationships in action unit intensities that have to be taken into account to improve data to be subsequently used for facial expression analysis. For this reason, in the proposed algorithmic pipeline, the AU intensities are, at first, temporally smoothed by using Adaptive-Degree Polynomial Filters [45].

To computationally model the variable and complex dependencies that exist among intensities of multiple AUs, Bayesian Network (BN) was used [46]. Each node of the BN is an AU label, and the links and their conditional probabilities capture the probabilistic dependencies among AUs [47,48].

**Figure 2:**
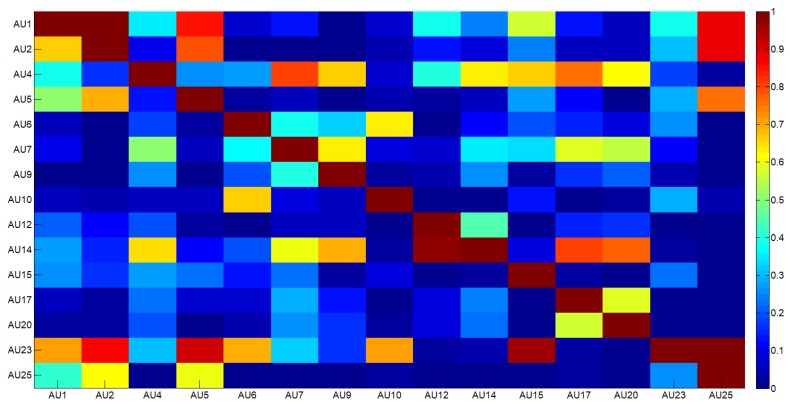
The learned joint probabilities among AUs.

**Figure 3:**
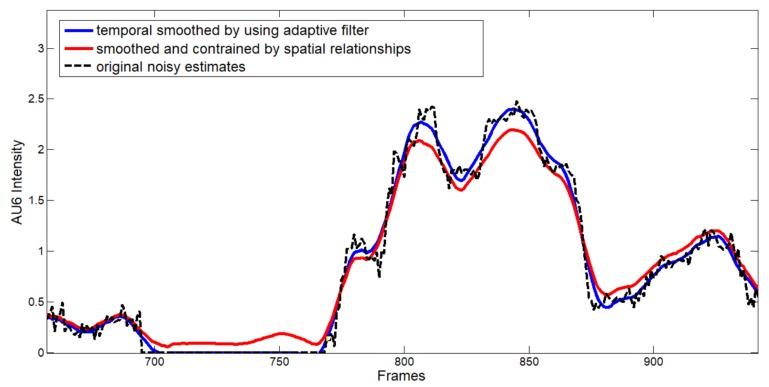
An example of regularized estimates for AU6.

AU dependencies were learned on the well known Cohn–Kanade Dataset (CK+) [49] and outcomes are reported in Figure 2, whereas Figure 3 shows an example of how the regularization step works: dotted black line is referred to the original estimates for AU6, blue line indicates the smoothed values obtained by using temporal adaptive filtering and, finally, red line refers to the estimates regularized by considering spatial constraints.

Starting from joint probabilities reported in Figure 2, the following regularization rule was applied:

$$\bar{AU_{i}}\left(t\right)=AU_{i}\left(t\right)+R_{i}\left(t\right)\;\forall i\in \left\{1\ldots n\right\}$$

where

$$R_{i}\left(t\right)=\frac{\sum_{j}\left(p_{i,j}\left(AU_{i}\left(t\right)-AU_{j}\left(t\right)\right)\right)}{n}\;\forall \left\{i,j\right\}\in \left\{1\ldots n\right\};i\neq j$$

with *n* the number of considered co-occurrent action units (in this paper n=14).

The output of this step are regularized AU intensities, i.e., AU intensities whose values at each time instant have been “smoothed” or “enhanced” depending on the values of the intensities of the related AUs in the joint CRF model.

It is worth noting here that studies of evolutionary nature have shown that the expression of emotions is universal and biologically determined (authors and dates). Autistic individuals have only a limited range of facial expressions [50] and this implies that learned AU dependencies keep valid for both typically and atypically developing/developed persons.

Finally, Facial Expressions Analysis can be performed on the basis of the estimated, smoothed and regularized AU intensities. In other words, the problem becomes how to relate the anatomically-based descriptors, i.e., the Action Unit intensities, with relevant facial expressions. The Facial Action Coding System (FACS) [51,52] is a comprehensive system for describing facial movements alone and in thousands of combinations can account for nearly all-possible facial expressions. The relationship between action units and facial expressions have been largely studied by psychologists [53] but also computationally evidenced by recent studies [54,55,56].

In the psychological scientific literature, the number of basic emotions is ambiguous (see [57]): some authors, including Ekman [58], attribute the character of fundamental emotions to happiness, sadness, fear, and anger; Johnson-Laird and Oatley added disgust [59]; and others [60] describe seven primary emotions adding interest and surprise. In this study, the Ekman model was taken as a theoretical reference, which is also the one followed by the most recent psychological tools for assessing children’s emotions (e.g., Test of Emotion Comprehension (TEC) [61]).

According to this, in this paper, the 14 facial action units that incorporate the most significant variations of eye brows, eye lids, cheeks and lips have been retained, ensuring this way to highlight the execution of the above four basic facial expressions. Table 1 reports the list of the facial action units considered in this paper together with a visual example of each of them and the corresponding facial expression in which each of them is involved in (H = Happiness; S = Sadness; F = Fear; and A = Anger).

On the one hand, since AU intensities are influenced by both non-emotional face configurations and movements, they cannot be directly used to quantify facial expression production. On the other hand, learned model do not apply correctly since datasets have a non-representative ratio of the desired class of interest and then biases in performance arise [62]. In this application context, it is in fact very difficult to provide labeled examples that can uniformly span over the huge and unpredictable range of data in input (related to personal facial cues, contextual executions, physical and mental disabilities as well as age groups). Besides, it is not straightforward to get a statistics of neutral face to be used as a computational baseline for two reasons: first, it is common practice in assessment or rehabilitation session to have many facial expressions in a short period of time and, second, non-emotional face configurations can change over time due to external or intrinsic factors such as level of engagement/fatigue or stereotyped movements.

To overcome the above drawbacks, in this paper, a statistic approach is used to quantitatively evaluate the facial expressions: at each time instant *t*, the variation in each AU intensity is computed by introducing a short-term statistics on a modeling window $W_{m}\left(t-\Delta_{m};t-1\right)$ where Δ is the observation period whose length depends on the expected temporal distance between two consecutive relevant facial expressions. The modeling window $W_{m}\left(t-\Delta_{m};t-1\right)$ is exploited to build a probabilistic model with multiple Gaussian functions built on the observed configurations of the facial muscles. In the obtained model, the probability to observe the value *X* of the intensity of AUi may be computed as:

$$P\left(X\right)=\sum_{i=1}^{K}w_{i}*\eta \left(X,\mu_{i},\sum_{i}\right)$$

where *K* is the number of distributions (K=3 in this paper), $w_{i}$ is an estimate of the weight of the *i*th Gaussian in the mixture, $\mu_{i}$ and $\sum_{i}$ are the mean value and covariance matrix the of the *i*th Gaussian, respectively, and η is a Gaussian probability density function.

Given the model, the largest value of AUi in the observation window $W_{o}\left(t+1;t+\Delta_{o}\right)$ is extracted, its probability to fit the model is computed and its negative log-likelihood

$$V_{AUi}\left(t\right)=-log(PDF\left(max\left(AUi\left(t\right):t\in W_{o}\right)\right)$$

is retained as a measure of the variation of the current values with respect to the expected ones.

It is important to observe that this way to proceed allows the system to get information about how the individual modifies his facial muscles by taking into account his facial features, the starting configuration of the face at the moment in which the facial expression is required and eventual stereotyped movements. In other words, what it is accounted for, is not the distance from a generic model but, the facial muscle variations before and after a request to produce a specific facial expression making the outcomes independent from eventual perturbed facial features (which is not unlikely to find in case of neurological diseases) or contextual circumstances.

These estimates will be finally used to compute a metric relative to each facial expression to be analyzed.

**Figure 4:**
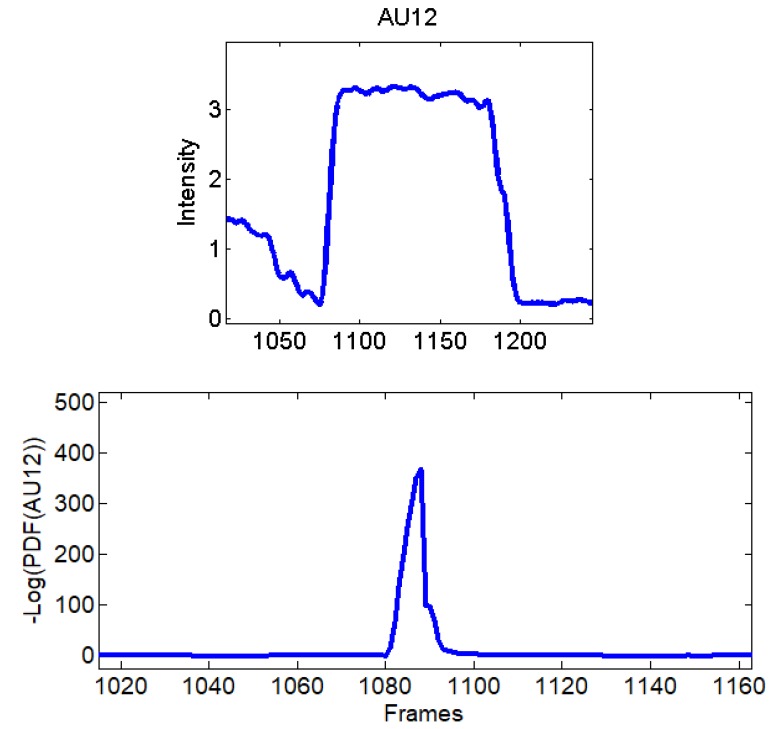
(**Top**) Intensity of AU12 while lip corners were pulled up; and (**Bottom**) corresponding computed variations.

Before going into details of the subsequent step, Figure 4 reports an example of computed variations for AU12 ($W_{m}=4$ s, $W_{o}=1$ s) starting from action units intensities extracted while lip corners were pulled up.

According to Table 1, for each of the four basic facial expressions considered, in each time instant facial expression production ability is separately computed for lower and upper face parts (indicated by uf and lf subscripts) as reported in Table 2.

Head pose and eye gaze estimation are used to control the reliability of action unit estimates and, consequently, of the whole facial expression analysis process. In particular, they are used to detect unreliable measurements, i.e., to discard outcomes of the whole pipeline in Figure 1 achieved when head positioning have a deviation from the frontal view greater than 30 degrees for one of three reference angles (pitch, yaw and roll). In each frame, head pose is estimated by using a 3D representation of facial landmarks and by projecting them to the image using orthographic camera projection (in absence of camera calibration parameters, a rough estimate based on image size is carried out). To control the uncertainty about the quality of the pose obtained as described above, eyes’ positions are extracted by using differential geometry and local self-similarity matching [63] and the found eye locations are used to get an independent new head pose vector. This eye based head pose vector is then compared with the one obtained by the 3D representation of facial landmarks and when the distance between the two pose vectors is larger than a certain threshold the vectors are averaged in order to get a more precise estimation of the head pose.

## 3. Assessment on a Publicly Available Dataset

To give evidence of the correlation between numerical outcomes of the proposed pipeline and individual abilities to perform facial expressions, a preliminary assessment on a sequence dataset containing persons with normotypic psychological and physical development, either while performing or not facial expressions, was performed. This gives a general significance to the system’s outcomes, making them self coherent, i.e., ready to supply valuable information even after a single acquisition session. To this aim, in this experimental phase, the image sequences of the Extended Cohn–Kanade Dataset (CK+) [49] were evaluated by the proposed pipeline described in Section 2. Among the 593 facial videos of the CK+ database, 327 of them are labeled and classified into one of the following categories: anger, disgust, fear, happiness, neutral, sadness, and surprise. The number of frames per video varies from 10 to 60, where the facial expression progressively changes, for each video, from the neutral frame to the apex expression frame. Considering the short duration of each video, processing was carried out after having set the modeling window $W_{m}$ to a quarter of the video length and the observation window $W_{o}$ to the length of the video minus the modeling window. At first, 20 sequences that do not contain any evident facial expression were processed and it was possible to observe that both the scores $M_{uf}^{x\in [HSFA]}$ and $M_{lf}^{x\in [HSFA]}$ remained lower than the value of 100 for all the sequences. This preliminary result makes it possible to identify that value (i.e., 100) as the lower bound of expressiveness, meaning that in case of lower outcomes it can be assumed that the related facial part does not produced any expression. Then, the 167 image sequences containing Happiness (69), Sadness (28), Fear (25) and Angry (45) facial expressions were processed by the algorithmic pipeline. By correlating scores and image sequences, it was possible to observe that in case of strong expressions corresponding system’s outcomes were always greater than 500, whereas in case of subtle expressions related system’s outcomes fell in the range [100;500].

Table 3 reports a summary of the scores obtained for subjects in the CK+ dataset annotated as performing facial expressions.

**Figure 5:**
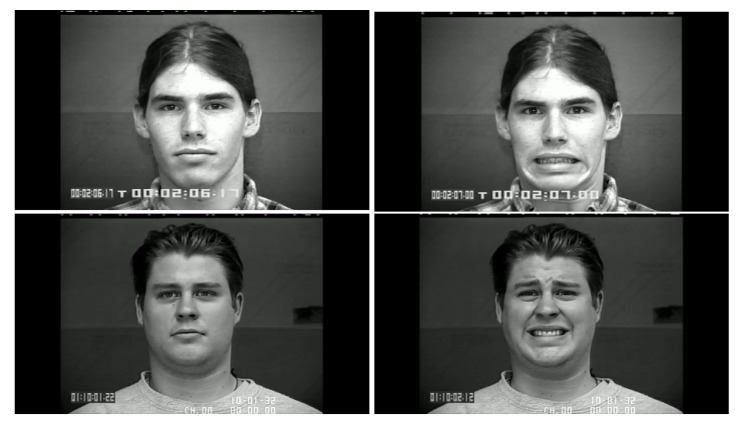
Two examples of fear execution in the CK+ dataset. Computational scores pointed out the expression performed by the Subject 54 (**first row**) is quantitatively worst than the one performed by the Subject 132 (**second row**).

It is worth noting that in Table 3 for a few sequences labeled as containing a facial expression, the system experienced very low scores. By analyzing in depth those unexpected numerical occurrences, it was possible to find out that they were obtained on the shortest videos, which contain only a very quick execution of the expression (making this way unreliable the statistical modeling). After this additional analysis, they were considered as outliers and not considered for the purposes of this preliminary experiment. Going into numerical details of computational outcomes, some additional interesting considerations arise. For example, the outcomes of the proposed approach make evident some flaws in producing facial expressions. In Figure 5, the first and last frame of the execution of fear expression by Subject 54 (sequence S054_002) and Subject 132 (sequence S132_003) are reported. The executions similarly got high scores for lower face part but very different scores for upper face part. Subject 132 got high score also for upper part whereas Subject 54 got a low score (66.1724). Although the productions qualitatively seem both well executed, the computational analysis gave numerical evidence that the Subject 54 actually does not significantly modify the eye regions making his execution quantitatively worst than other one. In our opinion, this could be very useful for selecting the best executions in the existing datasets (to be used for instance during training sessions), or to support the creation of new ones. Summing up, after the above experimental phase on the CK+ database it was possible to discover the existing relationships between the numerical outputs of the proposed pipeline and the individual ability in the production of facial expressions. Emerged numerical boundaries in computational scores make it possible to evaluate personal abilities in producing facial expression even during a single acquisition session. In fact, depending on the scores, it is possible to label the production ability of the subject under observation as “no ability” if the associated scores are under the detected lower bound of 100, “moderate ability” if the scores are in the range [100;500] and “strong ability” if the scores are greater than 500.

Finally, since the proposed pipeline could also be exploited to recognize if a facial expression is present or not in a given image sequence, it could of interest to point out the recognition performance on all the labeled sequences (167 containing one facial expression and 20 without facial expression) after setting the decision threshold to 500 for both lower and upper face parts. In the used detection and classification scheme, for each expression, if just one of the two scores (for upper and lower face part) was lower than the threshold the related expression was considered as not present. Otherwise, if both scores were greater than the threshold, the expression was considered as present. In the case in the same sequence two expressions were considered as present, the one with the larger average on the two scores was considered as prevalent.

The achieved Facial Expression Recognition (FER) results are reported in Table 4 where N stands for Neutral (no expression), whereas H, S, F and A stand for Happy, Sad, Fear and Anger expressions, respectively. Results can be can summarized as True Positive = 141/167 = 85%, False Positive = 2/20 = 10%, True Negative = 18/20 = 90% and False Negative = 25/167 = 15% that are not so far from the leading approaches in the literature [5] even if the proposed pipeline was not conceived for such FER purposes. It is worth noting that most of the false negative values correspond to those in the first column in Table 3, i.e., they are relative to very short videos which start immediately with a very quick onset.

Summing up, this experimental phase demonstrated the congruity of the numerical results with actual facial expression dynamics allowing therefore to proceed towards the key experimental step involving ASD children and aiming at quantitatively evaluating their skills in producing basic facial expressions (see next section).

## 4. Results on a Group of 17 ASD Children

The proposed pipeline was then tested on a group of 17 children with ASD diagnosis. Participants were recruited thanks to the collaboration with two non-profit Italian associations (“Amici di Nico Onlus” and “L’ Adelfia”) which offer intervention programs for children with ASD diagnosis and/or other disorders. The L’ Adelfia Ethics Committee gave approval for this study and informed signed consent was obtained from parents. The proposed pipeline was then tested on a group of 17 children (14 boys) with ASD (Autism Spectrum Disorder) diagnosis aged 6–13 years (Mean = 8.94; Standard Deviation = 2.41) without cognitive delay. Colored Progressive Matrix [64] were administered by trained psychologists to test if participants showed any cognitive delay. Results demonstrated that they had no mental retardation and their QI scores were on average level (Range: 90–120; Mean = 105; Standard Deviation = 10.98). Two children were twins, one child had a younger brother with ADHD diagnosis, and one had an older brother with a motor coordination disorder. All participants followed a behavioral intervention program using the Applied Behavioral Analysis (ABA). For the evaluation of the basic emotions production skills, an ad-hoc program was applied (see [65]). The administration occurred in a quiet room appropriately equipped for the child and trained psychologist assessed every participant in the presence of a familiar therapist.

**Figure 6:**
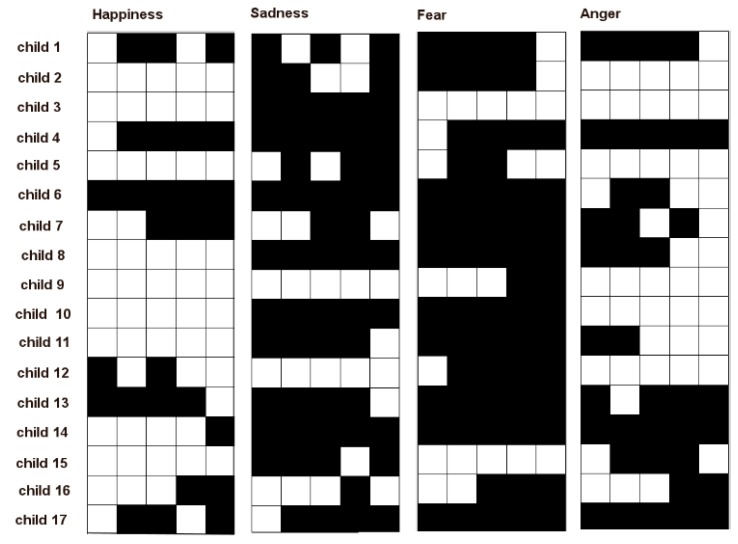
Annotations provided by a group of three expert professionals.

Each child was acquired while seated in front of an adult who ask him to produce one of the four aforementioned basic facial expressions. The requests of production of the facial expressions were provided sequentially to the child as happiness-sadness-fear-anger and the sequence was repeated five times. This way each child was asked to produce 20 facial expressions. A video was acquired for each child containing the whole session so at the end of the acquisition phase 17 videos were available. Each video has a different duration (minimum 2 min, maximum 4 min) depending on the degree of collaboration of the child and then on time spent to attract his attention at the beginning or even between one request and another. The requests were provided to the child with a minimum interval of 4 s from each other. The processing was performed by using a modeling window of 2 s and an observation window of 4 s. Videos were acquired from an off-the-shelf camera (image resolution 1920 × 1080 pixels, 25 fps) and each video was accompanied by information regarding the 20 time instants in which the requests were provided to the child. The recorded videos were independently scored by two psychologists (AL and FD) who annotated, for each request, if the child produced or not the correct facial expression that he/she was asked to produce. The consistency between the two coders was high (0.89). To reach full consensus, a third psychologist (FL) examined all videos, arbitrated, reasoned the discordance, and made final decisions. In Figure 6, the annotations made by the psychologists are reported. Each table is relative to a different facial expression (indicated on top of tables) and in each table the five columns correspond to the times each child was asked to perform the same facial expression. Each row is related to a child. A white cell indicates that the child correctly performed the facial expression, whereas a black cell indicates that the child did not perform the facial expression.

Acquired video sequences were subsequently processed by the proposed algorithmic pipeline.

This way, for each child and for each facial expression x=[HSFA], a score for upper $M_{uf}^{x}\left(t\right)$ and lower $M_{lf}^{X}\left(t\right)$ face was computed in each time instant *t* as described in Section 2.

**Figure 7:**
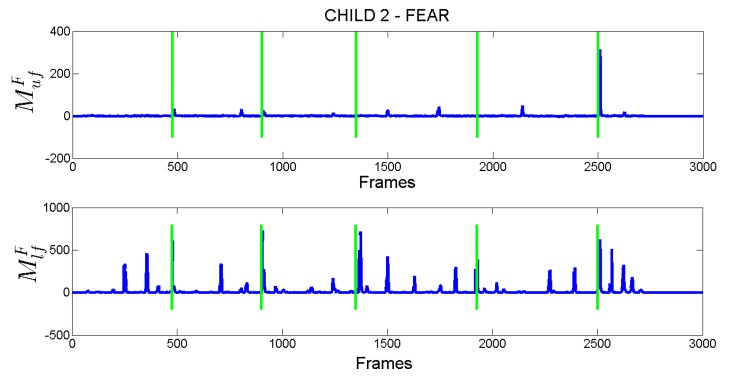
Measures of facial expression production ability for ASD Child 2 separately plotted for upper (**top**) and lower (**bottom**) face parts.Vertical green lines indicate the time instant in which the child was asked to produce the fear facial expression.

As an example, in Figure 7, the computed $M_{uf}^{F}$ and $M_{lf}^{F}$ scores for Child 2 are reported. Vertical green lines indicate the time instant in which the child was asked to produce the fear expression. Observing the plots in figure (according also to the annotations in Figure 6) is possible to derive that the child correctly produced the fear expression only on the last request (whose the request was made approximately in frame 2500) where both measures related to upper (sub-figure on top) and lower (sub-figure at the bottom) facial parts show high values in the time interval following the request (the expected time interval in which the child can produce the facial expression was set to 4 s). It is worth noting that after the previous four requests the child did not correctly produced the fear expression since, as rightly numerically highlighted in figure, he opened the mouth but he was not able to modify facial muscles related to the upper face and then to increase the intensity of related action units with respect to the state of his face at the moment of the request. This results in a $M_{uf}^{F}$ that presents low values after each of the first four requests.

**Figure 8:**
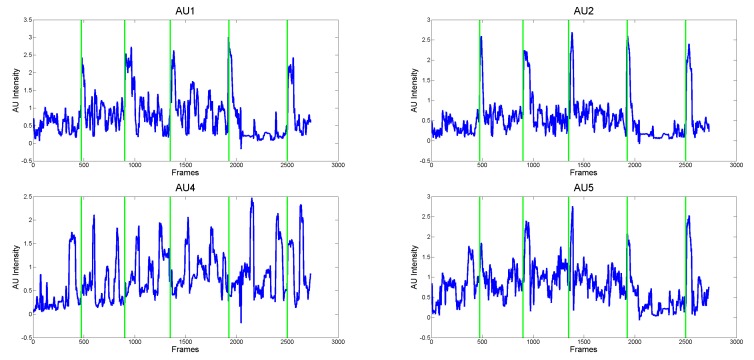
Intensity values computed for Child 2 for the action units involved in the production of the Fear expression and related to the upper face part (from left to right, top to bottom AU1, AU2, AU4 and AU5).

To better understand the benefit of using the proposed measures instead of the action unit intensities, in Figure 8, the intensity values for the AUs involved in the production of the Fear expression (i.e., AU1, AU2, AU4, AU5) for Child 2 are shown. It is straightforward to observe that the child continuously activated facial muscles (especially those related to the AU5) independently from the requests made by the person in front of him. This results in very cluttered signals in which many occurrences of AU activations arose, which could drive to the wrong conclusion that the child correctly performed all the required expressions. As shown in Figure 7, the proposed approach instead, is able to model these facial dynamics and then to identify when the child actually changes his expression following a specific request with respect to unsolicited facial movements (possibly due to stereotypes that are very common in ASD individuals).

**Figure 9:**
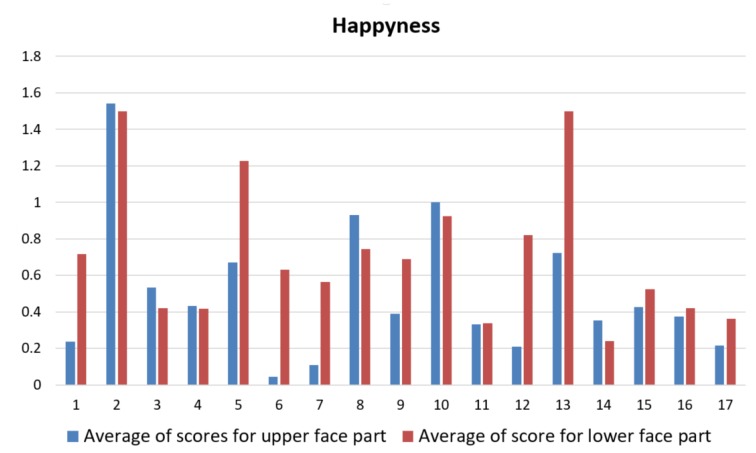

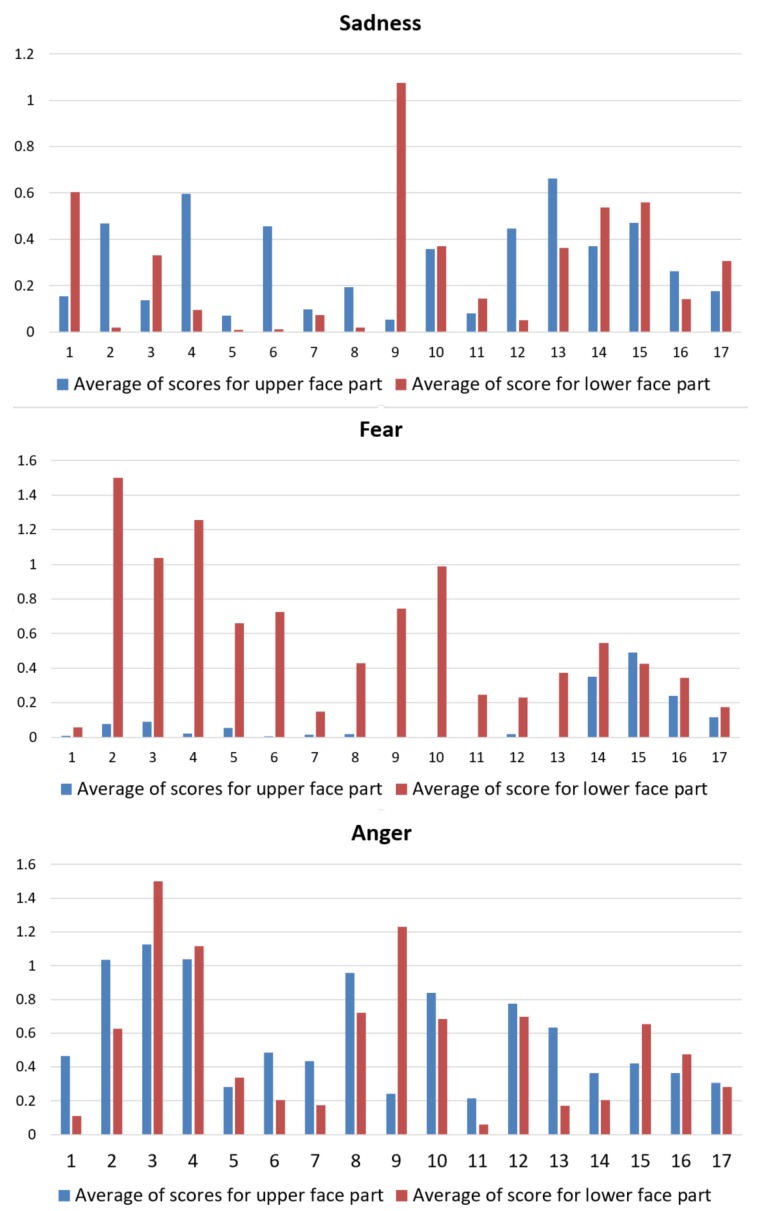
Graphical representations of average production scores computed for each of the 17 ASD children. From top to bottom: Happiness, Sadness, Fear and Anger related values.Blue bars are related to upper face part, whereas red bars are related to lower face part.

In Figure 9, the graphical representations of the average scores (on the five measurements corresponding to the five requests) computed for each of the 17 ASD children are reported. It is possible to observe that the children had higher difficulties to rightly produce the upper face configuration in producing fear expression (average score among children was ${\bar{M}}_{uf}^{F}=89.1341$) and the lower face configuration in producing sadness expression (${\bar{M}}_{lf}^{S}=276.8870$). As expected, the highest average score was computed instead for the lower face configuration while producing happiness expression (${\bar{M}}_{lf}^{F}=708.1951$).

This is the first evidence of the effectiveness of the proposed computational method for the estimation of the production skills of facial expressions, as it is possible to quantify the skills also being able to distinguish the abilities of individual children and even in their different parts of the face ([66,67]).

Another evidence of how the results of the proposed approach can be exploited can be obtained by comparing its computational outcomes with psychologists’ annotations. It is worth noting that children having the same qualitative behavior in the annotation tables in Figure 6 got instead quite different quantitative scores trough the proposed pipeline. As an example, consider the abilities of Child 2 and Child 3 to produce facial expression of happiness. In Figure 6, it is possible to derive that both children obtained five correct executions according to the expert observations. However the scores computed by the proposed pipeline demonstrated that the ability of Child 2 is much greater than the one of Child 3, since his scores for happiness production were about three times greater than those obtained by Child 3.

**Figure 10:**
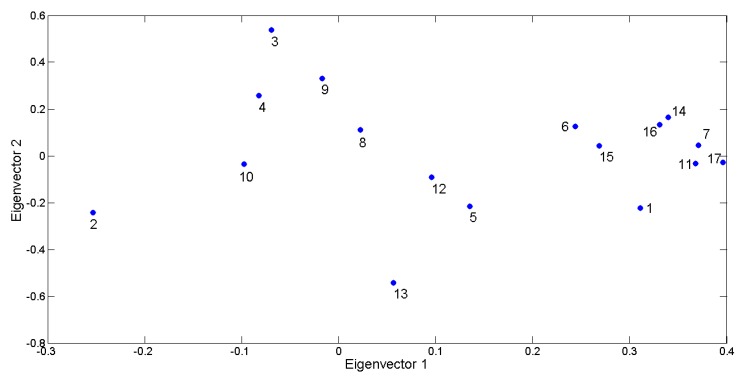
2D visual representation of the data variability in the computed scores for the 17 ASD children.

To provide information about the global variability represented by the outcomes provided by the proposed approach, the scores produced for each child have been put in a matrix having size [17 × 40] and then a distance matrix between the 17 children has been calculated by using Euclidean distance. Then, principal directions have been computed as the eigenvectors of the covariance matrix as well as the eigenvalues that represent the percentage of captured variability of data. The 2D visual representation of the 17 ASD children has been produced and reported in Figure 10 where close to each marker the ID of the related child is placed. The plot represents the data spreading on the first two principal components that retained almost the 50% of data variability (PCA1 37%, PCA2 13%). Figure 10 points out that the computed scores are able to take into account the differences and similarities in producing facial expressions. In other words, this plot demonstrates as children with similar abilities have points close in this distance space. This can be better realized by comparing the plot with graphical representations in Figure 9. For example, the plot shows that Child 2 has a point in the plot very distant from the other children. In fact, as previously stated, Child 2 showed the greatest ability in the sample group in producing happiness expression and, on the contrary, he was among the worst in producing sadness expression. Similarly, Child 13 got the lowest coefficient for eigenvector 2 since he was the best one in producing anger expression, whereas he was among the worst ones in producing remaining expressions. These abilities so selective, very good for an expression and very poor for the others, make the aforementioned children very different from the others and this is well highlighted by the graph. Accordingly, Children 7, 17, and 11 have very close points associated because they were, more or less to the same extent, unable to properly produce any facial expression.

On the one hand, this accurate quantification of production capacities can allow caregivers and physicians to better understand the behavior of children and, possibly during different sessions, their correlation with external factors such as fatigue, adult behavior, environment, etc. On the other hand, it can also tell the professionals what aspect of production should be emphasized while providing the subsequent therapies.

Summing up, the proposed approach can be used as a magnifying glass which can further allow professionals to clearly distinguish which skills in the production of facial expressions are more compromised and then provide a unique indication to program targeted interventions.

**Figure 11:**
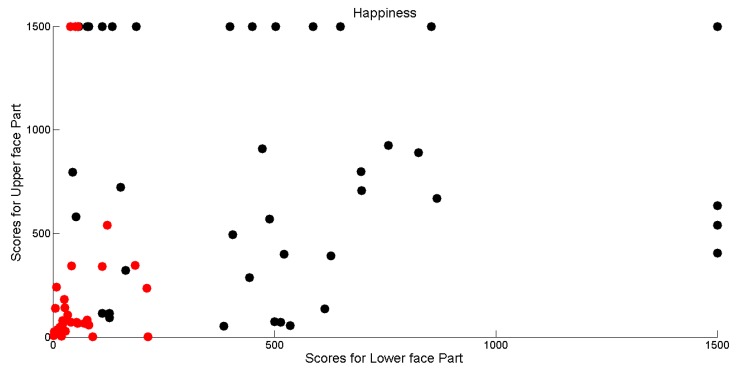

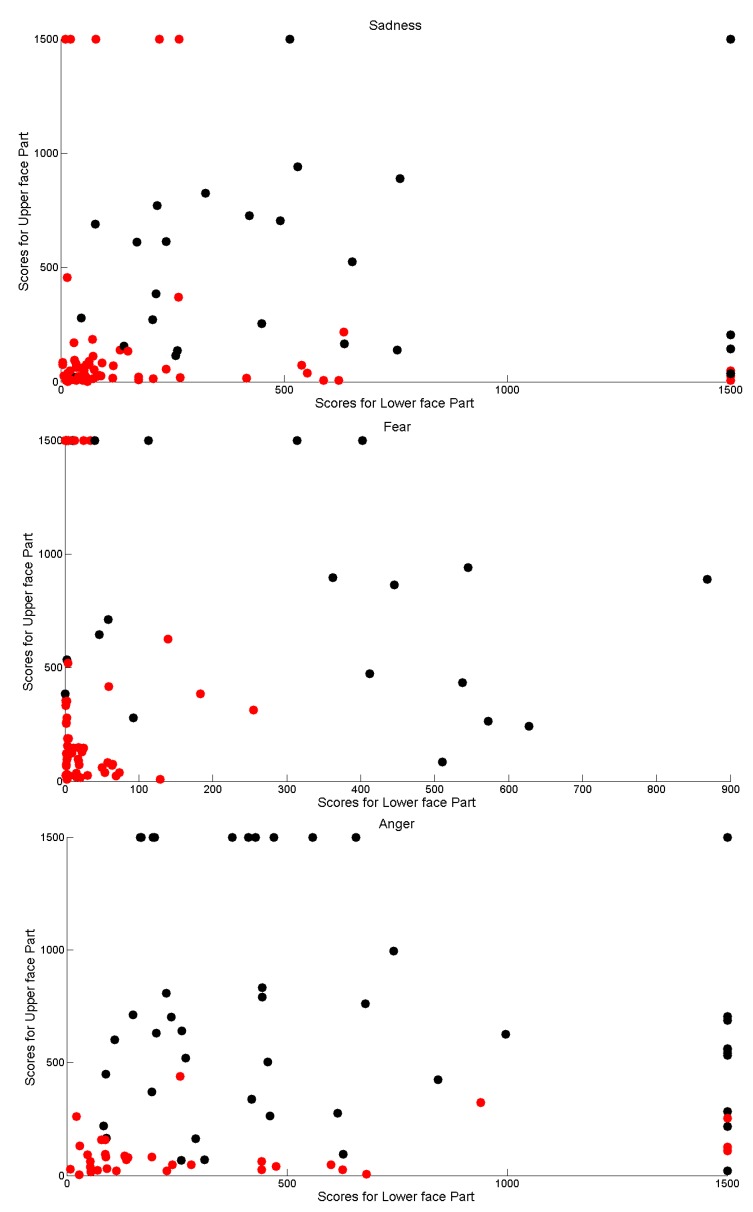
Plot into the $M_{lf}^{H}$ (Happiness—lower face measure)-$M_{uf}^{H}$ (Happiness—upper face measure) plane of the dispositions of measures for Happiness production in the 17 ASD children.

This is much more evident while deeply correlating computational outcomes with the manual annotations performed by the group of psychologists. In Figure 11, the gathered scores for each of the four facial expressions have been plotted into the $M_{lf}^{x}$ (measure related to the lower face part for expression x∈{H,S,F,A})–$M_{uf}^{x}$ (measure related to the upper face part for expression *x*) plane. Red points correspond to measure for executions that were annotated as “facial expression not produced”, whereas black points correspond to measure for executions that were annotated as “facial expression produced”. Greatest values have been bounded to 1500 in order to improve graph readability.

It is possible to observe that in most cases high scores correspond to positive annotations. However, it is interesting that sometimes psychologists were misled by different behaviors exhibited by the lower and upper part of the face. This is evidenced by the positive annotation relative to very low scores for upper or lower part (black points very close to the horizontal or vertical axis).

Another relevant consideration is that in each plot there is an “uncertainty” area in which psychologists annotated as positive or negative occurrences with very similar computational outcomes. The boundary of this area depends on the expression and, in general, it leads towards the vertical axis since, by observing the videos, it is much more harder to detect movement in the eye regions with respect to those of the mouth region. This is not surprising since there are some relevant studies that explain the difficulty in judgment by pointing out the frequent errors people commit due to perceptual or attentional factors that undermine the detection process of facial movements. They argued that difficulties in discriminating may arise from an omission of or inattention to authenticity cues by human observers, because such cues are subtle and infrequent in most cases. Nevertheless, the limitations of perceptual and attentional mechanisms (perceptual-attentional limitation theory) remains a subject of debate [29] and the only road currently practicable to overcome them is to rely on systems based on computational methods such as the one proposed in this article.

## 5. Conclusions

This paper presents a processing pipeline based on computer vision methods able to analyze facial expression in unconstrained conditions. The pipeline provides computational outcomes able to quantify in a personalized manner the ability to produce facial expressions independently from stereotypes or non-emotional face configurations. After a preliminary assessment on the CK+ dataset, the pipeline was exploited to analyze the facial expression production ability of 17 ASD children demonstrating how it allows stepping up from a qualitative binary evaluation provided from a group of psychologists to a quantitative and numerically continuous description that could help to go beyond the limitations of traditional ASD assessment protocols, allowing the professionals to plan targeted therapies. A limitation of the present study is the sample size. The number of participants should be evaluated considering the low prevalence of the autism disorder, 1 on 59 children aged 8 years old [68]. Besides, it should taken into consideration that the participants were chosen on the specific range of chronological age for the administration of the Test of Emotion Comprehension (TEC), which limited even more the size of the population. Notwithstanding, future research should replicate the training to sustain the generalization of our results to the population of children with ASD. Future works will deal also with the monitoring of the evolution of children’s skills over time to objectively highlight the improvements, for example by comparing the individual ability to produce specific facial expression before and after targeted therapies. Finally, the possibility to exploit computer vision algorithms for motion magnification to give professionals a visual feedback of numerical outcomes will be investigated.

## Figures and Tables

**Table 1:** Action Units used for monitoring the ability in producing the foru basic facial expressions (H = Happiness; S = Sadness; F = Fear; and A = Anger).

AU	Full Name	Example	Involved in
AU1	Inner brow raiser	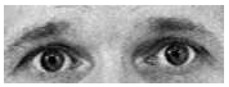	S-F
AU2	Outer brow raiser	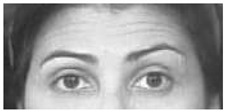	F
AU4	Brow lowerer	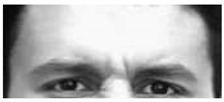	S-F-A
AU5	Upper lid raiser	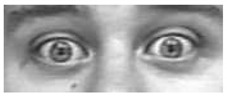	F-A
AU6	Cheek raiser	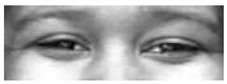	H
AU7	Lid tightener	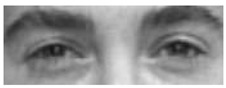	A
AU9	Nose wrinkler	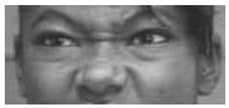	A
AU12	Lip corner puller	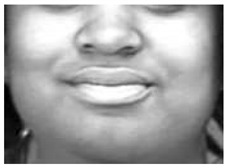	H
AU15	Lip corner depressor	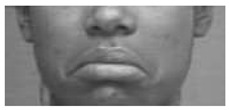	S
AU17	Chin raiser	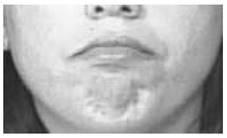	A
AU20	Lip stretched	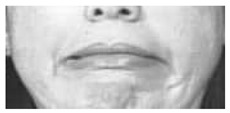	F
AU23	Lip tightener	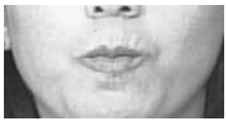	A
AU25	Lips part	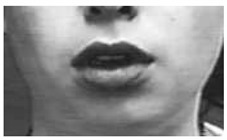	A
AU26	Jaw drop	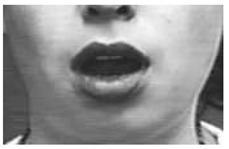	F

**Table 2:** Measure computation of production ability for: H = Happiness; S = Sadness; F = Fear; and A = Anger. Time index has been omitted for a better table readability.

Facial Expression	Production Scores
H	$M_{uf}^{H}=V_{AU6}$
	$M_{lf}^{H}=V_{AU12}$
S	$M_{uf}^{S}=max(V_{AU1},V_{AU4)}$
	$M_{lf}^{S}=V_{AU15}$
F	$M_{uf}^{F}=min\left(max\left(V_{AU1},V_{AU2}\right),V_{AU4},V_{AU5}\right)$
	$M_{lf}^{F}=max\left(V_{AU20},V_{AU26}\right)$
A	$M_{uf}^{A}=max\left(V_{AU4},V_{AU5},V_{AU7}\right)$
	$M_{lf}^{A}=max\left(max\left(V_{AU9},V_{AU23}\right),min\left(V_{AU17},V_{AU25}\right)\right)$

**Table 3:** An overview of the scores obtained by the proposed pipeline on the subjects in the CK+ dataset.

	M<100	100≤M≤500	M>500
$M_{lf}^{H}$	4 (6%)	5 (8%)	60 (86%)
$M_{uf}^{H}$	2 (3%)	2 (3%)	65 (94%)
$M_{lf}^{S}$	4 (14%)	2 (7%)	22 (79%)
$M_{uf}^{S}$	2 (7%)	0	26 (93%)
$M_{lf}^{F}$	2 (8%)	0	23 (92%)
$M_{uf}^{F}$	3 (12%)	3 (12%)	19 (76%)
$M_{lf}^{A}$	2 (5%)	3 (7%)	40 (88%)
$M_{uf}^{A}$	0	5 (12%)	40 (88%)
overall	19 (11%)	20 (12%)	124 (77%)

**Table 4:** Facial Expression Recognition performance on the CK+ dataset (N = Neutral; H = Happiness; S = Sadness; F = Fear; and A = Anger).

	N	H	S	F	A
N	**18**	1	1	0	0
H	0	**68**	1	0	0
S	4	2	**19**	2	1
F	0	0	2	**21**	2
A	5	2	2	3	**33**
*Overall*	*90%*	*99%*	*67%*	*84%*	*73%*
